# Urinary Estrogen Metabolites and Self-Reported Infertility in Women Infected with *Schistosoma haematobium*

**DOI:** 10.1371/journal.pone.0096774

**Published:** 2014-05-21

**Authors:** Júlio Santos, Maria João Gouveia, Nuno Vale, Maria de Lurdes Delgado, Ana Gonçalves, José M. Teixeira. da Silva, Cristiano Oliveira, Pedro Xavier, Paula Gomes, Lúcio L. Santos, Carlos Lopes, Alberto Barros, Gabriel Rinaldi, Paul J. Brindley, José M. Correia da Costa, Mário Sousa, Mónica C. Botelho

**Affiliations:** 1 Clínica da Sagrada Esperança, Luanda, Angola; 2 CIQUP, Chemistry and Biochemistry Department, Faculty of Sciences, University of Porto, Porto, Portugal; 3 INSA, National Institute of Health, Porto, Portugal; 4 Centre for Reproductive Genetics Prof. Alberto Barros, Porto, Portugal; 5 Experimental Therapeutics and Pathology Research Group, Portuguese Institute of Oncology, Porto, Portugal; 6 Department of Pathology and Molecular Immunology, Institute of Biomedical Sciences Abel Salazar (ICBAS), University of Porto, Porto, Portugal; 7 Department of Genetics, Faculty of Medicine, University of Porto, Porto, Portugal; 8 Department of Microbiology, Immunology and Tropical Medicine, and Research Center for Neglected Diseases of Poverty, School of Medicine & Health Sciences, George Washington University, Washington, D.C., United States of America; 9 Departamento de Genética, Facultad de Medicina, Universidad de la República, (UDELAR), Montevideo, Uruguay; 10 Center for the Study of Animal Science, CECA/ICETA, University of Porto, Porto, Portugal; 11 Department of Microscopy, Laboratory of Cell Biology, Institute of Biomedical Sciences Abel Salazar (ICBAS), Multidisciplinary Unit for Biomedical Research-UMIB, University of Porto, Porto, Portugal; The George Washington University Medical Center, United States of America

## Abstract

**Background:**

Schistosomiasis is a neglected tropical disease, endemic in 76 countries, that afflicts more than 240 million people. The impact of schistosomiasis on infertility may be underestimated according to recent literature. Extracts of *Schistosoma haematobium* include estrogen-like metabolites termed catechol-estrogens that down regulate estrogen receptors alpha and beta in estrogen responsive cells. In addition, schistosome derived catechol-estrogens induce genotoxicity that result in estrogen-DNA adducts. These catechol estrogens and the catechol-estrogen-DNA adducts can be isolated from sera of people infected with *S. haematobium*. The aim of this study was to study infertility in females infected with *S. haematobium* and its association with the presence of schistosome-derived catechol-estrogens.

**Methodology/Principal Findings:**

A cross-sectional study was undertaken of female residents of a region in Bengo province, Angola, endemic for schistosomiasis haematobia. Ninety-three women and girls, aged from two (parents interviewed) to 94 years were interviewed on present and previous urinary, urogenital and gynecological symptoms and complaints. Urine was collected from the participants for egg-based parasitological assessment of schistosome infection, and for liquid chromatography diode array detection electron spray ionization mass spectrometry (LC/UV-DAD/ESI-MSn) to investigate estrogen metabolites in the urine. Novel estrogen-like metabolites, potentially of schistosome origin, were detected in the urine of participants who were positive for eggs of *S. haematobium*, but not detected in urines negative for *S. haematobium* eggs. The catechol-estrogens/ DNA adducts were significantly associated with schistosomiasis (OR 3.35; 95% CI 2.32–4.84; *P*≤0.001). In addition, presence of these metabolites was positively associated with infertility (OR 4.33; 95% CI 1.13–16.70; *P*≤0.05).

**Conclusions/Significance:**

Estrogen metabolites occur widely in diverse metabolic pathways. In view of the statistically significant association between catechol-estrogens/ DNA adducts and self-reported infertility, we propose that an estrogen-DNA adduct mediated pathway in *S. haematobium*-induced ovarian hormonal deregulation could be involved. In addition, the catechol-estrogens/ DNA adducts described here represent potential biomarkers for schistosomiasis haematobia.

## Introduction

At least 243 million people are infected with schistosomes, and more than half of these cases are caused by *Schistosoma haematobium*, the causative agent of urogenital schistosomiasis [1], [2], [3]. Indeed the number of cases of *S. haematobium* may be much greater than previously believed - perhaps as many as triple that of earlier estimates of prevalence [4]. If confirmed, urogenital schistosomiasis may represent the most common neglected tropical disease in sub-Saharan Africa [5]. The adult stages of the blood flukes are long-lived within the venous plexi draining the pelvic organs including the urinary bladder, uterus, vagina, seminal vesicles and prostate [6]. The terminal spine eggs released continuously from the female schistosomes migrate from the circulation by breaking through the endothelial cells lining the venules. Thereafter, they traverse the wall of the urinary bladder to the lumen from where they exit to the exterior environment with the urine, to complete the transmission of this neglected tropical disease pathogen.

Problematically, many schistosome eggs fail to exit the body, and lodge with interstitial tissues of these organs. Entrapped eggs of *S. haematobium* induce granulomata and may be identified as various clinical presentations of ‘sandy patches’ and other signs and symptoms [3]. Hematuria is caused by the inflammation induced by entrapped eggs in the bladder and ureters. In addition, organ damage can follow obstruction of the ureters, as can secondary urinary tract and renal infections, hydronephrosis and renal failure [3]. Moreover, eggs of *S. haematobium* are biological carcinogens, grouped with a dozen or so other Group 1 microbes by the World Health Organization's (WHO) International Agency for Research on Cancer [7]. Chronic exposure to the eggs of *S. haematobium* frequently leads to squamous cell carcinoma of the bladder (SCC), the incidence of which is highest in regions endemic for *S. haematobium* [8], [9]. Indeed, SCC is one of the most serious complications of chronic schistosomiasis haematobia [4], [8], [9], [10], [11]. In addition, the schistosomiasis haematobia likely influences endocrine homeostasis based in part on its ability of the blood flukes to synthesize and release estradiol [12], [13].

**Figure 1:**
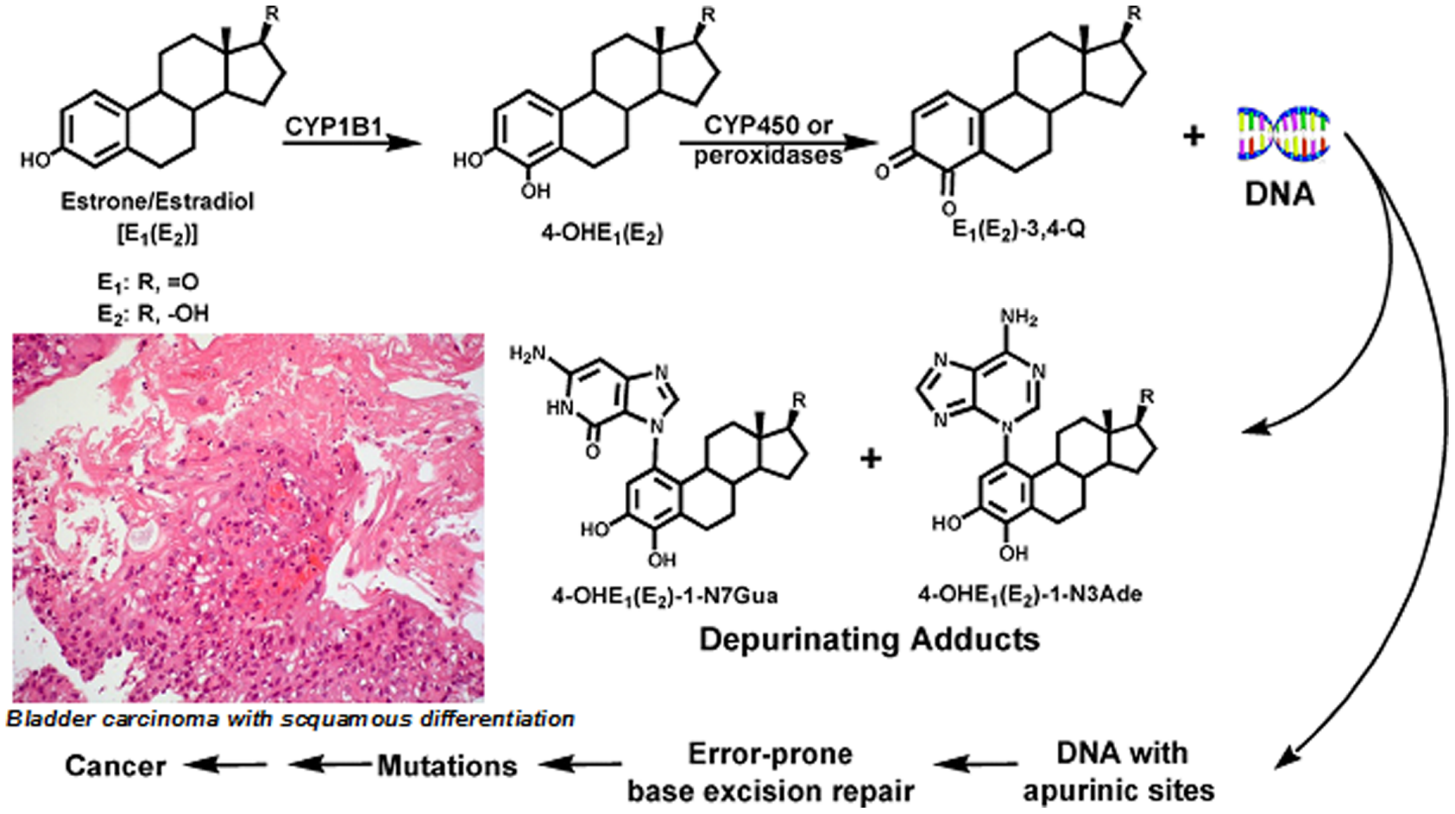
Major metabolic pathways in cancer initiation by estrogens. Adapted from [21], [22].

We reported that extracts of *S. haematobium* worms include estrogen-related metabolites that down-regulate estrogen receptors (ER) alpha and beta in estrogen responsive cells *in vitro* [13]. Further, we identified estrogen metabolites in *S. haematobium* eggs by mass spectrographic analysis and also detected these schistosome-derived estrogens in sera of *S. haematobium*-infected persons. These estrogen-like metabolites belong to the catechol-estrogen family of molecules [14]. Estrogens arise by aromatization of androstenedione and testosterone, catalyzed by cytochrome P450 (CYP) 19, aromatase. They are metabolized by two major pathways – (1) formation of catechol estrogens and (2) 16α-hydroxylation [15] (Fig. 1). The natural estrogens, estrone (E1), and estradiol (E2), are metabolized at the 2- or 4-position with the formation of catechol estrogens that, in turn, are metabolically oxidized into catechol estrogen quinones (CEQ). CEQ have been implicated in the etiology of cancers [16]; the interaction of CEQ, in particular CE-3,4-Q, with DNA produces depurinating adducts, which may generate DNA apurinic sites that, in turn, initiate mutations [17]. In the catechol pathway, the metabolism involves further oxidation to semiquinones and quinones, including formation of the catechol estrogen-3,4-quinone, the major mutagenic derivative of estrogen [18]. Estrogen-derived metabolites associated with carcinogenesis in diverse tissues including the breast and thyroid [18], [19], [20] might impact target cells either as hormones (hence affecting gene expression regulation) and/or as carcinogens that are directly genotoxic leading to formation of chromosomal DNA-adducts. Both mechanisms could be involved in carcinogenesis [20].

Given that the eggs of *S. haematobium* secrete novel catechol-estrogens, and that in other contexts, estrogens are metabolized to active quinones that modify DNA [14], we now hypothesize that infertility during infection with *S. haematobium* might be related to the presence of parasite-derived catechol-estrogens. To begin to address this issue, which may have substantial implications for global health, here we undertook a cross-sectional study of residents of rural Angola, a region endemic for schistosomiasis haematobia [21]. A significant association between the presence of catechol-estrogens/ DNA adducts in the urine of females who were urine egg-positive for *S. haematobium* infection and self-reported infertility was detected.

## Methods

### Study area

**Figure 2:**
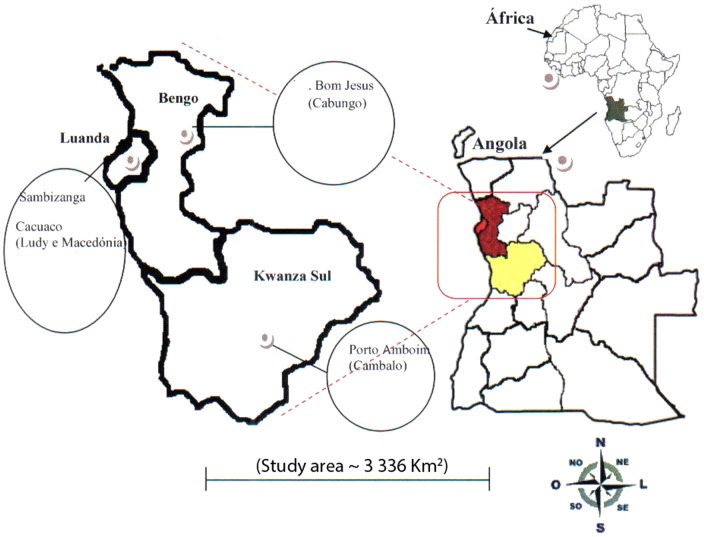
Map of Angolan study area. Adapted from [42].

Bengo province is a rural region, close to Luanda, the capital city of Angola. The study was performed in 2011 and 2012 at the Clínica da Sagrada Esperança, Luanda, Angola. This clinic serves the residents of Bengo. *S. haematobium* is endemic in this province; transmission occurs proximal to the Muxima River (Fig. 2). Bengo has a population of 500,000 inhabitants most of whom are involved with rural activities.

### Study population and design

Irrespective of symptoms, 93 females willing to provide a urine sample were enrolled in the study. The ages of these participants ranged from 2 to 93 years. All participants provided oral informed consent. They all were advised that were free to withdraw from the study at any time, without the need for an explanation. Oral informed consent was documented by the physician performing the interviews. Written informed consent could not be obtained because participants were illiterate. Parents provided informed consent on behalf of participating children. Ethical approval for the study protocol, including the use of oral consent, was obtained from the review board (the local ethics committee) of Clinica da Sagrada Esperança, Luanda, Angola. The 93 volunteers were arbitrarily assigned to three age categories: 2–11 years (children), 37 participants; 12–19 years (adolescents), 17 participants; and 20–94 years (women), 39 participants.

### Urine examination

Patients provided one sample of urine of ∼50 ml volume, collected at the time of the interview. The entire micturition volume was filtered through a polycarbonate membrane of 14 µm mesh size and 25 mm diameter (Whatman plc, Springfield Mill, UK). Thereafter the membrane was stained with Trypan blue (Sigma-Aldrich Corp, St. Louis, MO). Schistosome ova retained on the membrane were identified with the aid of a light microscope, as described [22].

### Medical history and clinical examination

Interviews were undertaken in a relaxed atmosphere. It was emphasized to the participants by the project leaders that the consultation was confidential and voluntary. Personal data were obtained in order to facilitate (subsequent) follow-up in the advent of subsequent appearance of cancer or other illnesses. Each participant was asked questions related to previous and present urinary system symptoms, gynecological complaints and general medical history and treatments. Regarding the fertility status, participants were assigned to one of three groups conforming to the WHO's definition of infertility: “Infertility is the inability of a woman to become pregnant after one year of sexual intercourse without using contraception, with primary infertility considered when the woman never conceived, and secondary infertility when she had had a previous labour” [23]. Thus, in accordance, groups were defined as those who reported absence of difficulties in becoming pregnant (Group 1); those who were unable to become pregnant after one year of trial (self-reported primary infertility - Group 2); and those who had previously borne one child but could not achieve a second child (self-reported secondary infertility - Group 3). An urologist (JS) performed the interviews.

### Liquid Chromatography Diode Array Detection Electron Spray Ionization Mass Spectrometry (LC/UV-DAD/ESI-MSn)

Analysis using LC/UV-DAD/ESI-MSn was performed on a Finnigan Surveyor Plus High-Performance Liquid Chromatography (HPLC) instrument (ThermoFinnigan, San Jose, CA) equipped with a diode-array detector and a mass detector. The HPLC system included a quaternary pump, autosampler, degasser, photodiode-array detector and automatic thermostatic column compartment, and was controlled by the Xcalibur software package (ThermoFinnigan). The mass detector was a Finnigan Surveyor LCQ XP MAX quadrupole ion trap mass spectrometer (ThermoFinnigan) equipped with an electron spray ionization (ESI) interface. Control and data acquisition were carried out with Xcalibur. Nitrogen gas of >99% purity was employed with a pressure of 520 kPa (75 psi). The instrument was operated in a negative-ion mode with an ESI needle voltage of 5.00 kV and capillary temperature of 325 °C. The full scan covered the mass range from 50–2,000 m/z (mass/charge number of ions). Mass spectrometry (MSn) data were simultaneously acquired for the selected precursor ion. Collision Induced Dissociation-Tandem Mass Spectrometry (CID-MS/MS) and MSn analyses were performed using helium with collision energy of 25–35 eV. The HPLC used a LiChroCART C18 column (125 mm×4 mm; 5 µm particle diameter, end-capped) with the temperature maintained at 25 °C; the mobile phase was composed of 1% (v/v) acetic acid in water (**A**) and acetonitrile, which had been degassed and filtered (**B**). The gradient used was 0–5 min with 100% **A**; 5–10 min, linear gradient from 100% to 80% **A**; 10–15 min, 80% **A**; 15–50 min, linear gradient from 80% to 40% **A**; 50–65 min, 40% **A**; 65–75 min, linear gradient from **A** to 100% **B**. The flow rate was 0.3 ml min^−1^ followed to split out in 200 µl min^−1^ to MS. Spectral data for all peaks were accumulated in the range of 200–600 nm. The instrument was calibrated with caffeine (Sigma-Aldrich), Met-Arg-Phe-Ala (MRFA) (tetrapeptide, Thermo Finnigan), and Ultramark 1621 (Lancaster Synthesis, Ward Hill, MA) in the mass range of 195–1,821 m/z. An advantage of inclusion of a MS detector over conventional HPLC analysis is that the number of channels in the detector can be set to specifically and separately identify all estrogen related compounds in a single injection of the urine sample.

### Statistical analysis

Groups -- schistosomiasis positive and negative, presence or absence of metabolites -- were compared using the chi-square test with Yate's correction or with Fisher's exact, two-sided test when expected values were below 5. The chi-square test was used because it compares categorical variables. The data were normally distributed. Differences were considered statistically significant where *P*≤0.05 (VassarStats, Poughkeepsie, NY).

## Results

### Association between self-reported infertility and the presence of *Schistosoma haematobium* eggs in the urine

All 93 participants had lived in the area for at least two years. Urinary *S. haematobium* infection was diagnosed when schistosome ova were identified in the urine. The participants were assigned to one of two groups: positive and negative for *S. haematobium* infection. The median age was 22.9 (range 6 to 94) years for *S. haematobium*-positive and 27.3 (2 to 88) years for *S. haematobium*-negative females (Table 1). The fertility status of the women, excluding the prepubescent population (2–12 years old), and the presence of *S. haematobium* eggs in urine revealed 24 fertile and 29 women with a history of self-reported infertility --14 in Group 2, i.e. unable to become pregnant after one year trial, and 15 in Group 3, i.e. had borne fewer children than desired (Table 2). In general, signs and symptoms reported by the participants were distributed similarly between the groups, *S. haematobium* +ve and *S. haematobium* –ve, including dysuria, lower abdominal pain, history of water contact, etc. (Table 3).

**Table 1:** Numbers of positive (*S. haematobium* +ve) and negative (*S. haematobium* -ve) urine samples for eggs of *Schistosoma haematobium* according to the age of the participants.

Age groups (years)	*S. haematobium* +ve	*S. haematobium* -ve	Total
median age (range)	22.9 (6–94)	27.3 (2–88)	25.1 (2–94)
2–11, i.e. children	18	19	37
12–19, i.e. adolescents	12	5	17
20–94	16	23	39
Total	46	47	93

**Table 2:** Status of fertility among Angolan women who provides urine samples positive (*S. haematobium* +ve) and negative (*S. haematobium* -ve) for eggs of *Schistosoma haematobium*, according to the age of the participants.

	N (%)	median age (years)	Age range (years)
***Non-reproductive females***	40 (100%)	8.25	2–12
*S. haematobium* +ve	21 (52.5%)	9.3	6–12
*S. haematobium* –ve	19 (47.5%)	7.1	2–11
***Fertile women (Group1)***	24 (100%)	51	18–94
*S. haematobium* +ve	8 (33.3%)	52.5	18–94
*S. haematobium* –ve	16 (66.7%)	50.25	19–88
***Self-reported infertility (Group 2+3)*** a	29 (100%)	26.9	17–41
*S. haematobium* +ve	17 (58.6%)	25.6	18–41
*S. haematobium* –ve	12 (41.4%)	28.8	17–40
***Self-reported primary infertility (Group 2)***	14 (100%)	18.8	17–21
*S. haematobium* +ve	10 (71.4%)	19	18–21
*S. haematobium* -ve	4 (28.6%)	18.3	17–19
***Self-reported secondary infertility (Group 3)***	15 (100%)	34.5	24–41
*S. haematobium* +ve	7 (46.7%)	35.1	27–41
*S. haematobium* -ve	8 (53.3%)	34	24–40

a Women had been unable to become pregnant after one year of trial (self-reported primary infertility, Group 2) and those who had borne fewer children than they desired (self-reported secondary infertility, Group 3).

**Table 3:** Symptoms and fertility state of 93 female residents of the Bengo region of Angola, an area endemic for schistosomiasis haematobia.

Symptom	*Sh egg* +ve; n = 46	*Sh egg* –ve; n = 47	OR	95% CI	*P*-value
Hematuria	35	22	3.9	1.6–9.8	≤0.01
Dysuria	26	23	1.43	0.63–3.25	0.27
Lower abdominal pain	24	22	1.3	0.57–2.95	0.34
History of water contact	37	39	1.1	0.38–3.14	0.54
Previous treatment with praziquantel	16	10	0.44	0.17–1.13	0.07
Estrogen metabolites	25	0	3.35	2.32–4.84	≤0.01
**Fertility status**					
Fertile	8/24	16/24	0.98	0.33–2.97	0.61
Group 2+3	17/29	12/29	1.02	0.34–3.07	0.60
Group 2	10/29	4/29	4.06	0.94–17.4	≤0.03
Group 3	7/29	8/29	0.4	0.10–1.40	0.12

Women unable to become pregnant after one year of trial (self-reported primary infertility - Group 2) and those who had borne fewer children than desired (self-reported secondary infertility - Group 3).

*Sh egg* +ve, positive for eggs of *S. haematobium* in urine; *Sh egg* –ve, negative for eggs of *S. haematobium* in urine.

OR, odds ratio; CI, confidence interval.

### Novel estrogen-like metabolites detected in urine of females positive for eggs of *S. haematobium*

**Figure 3:**
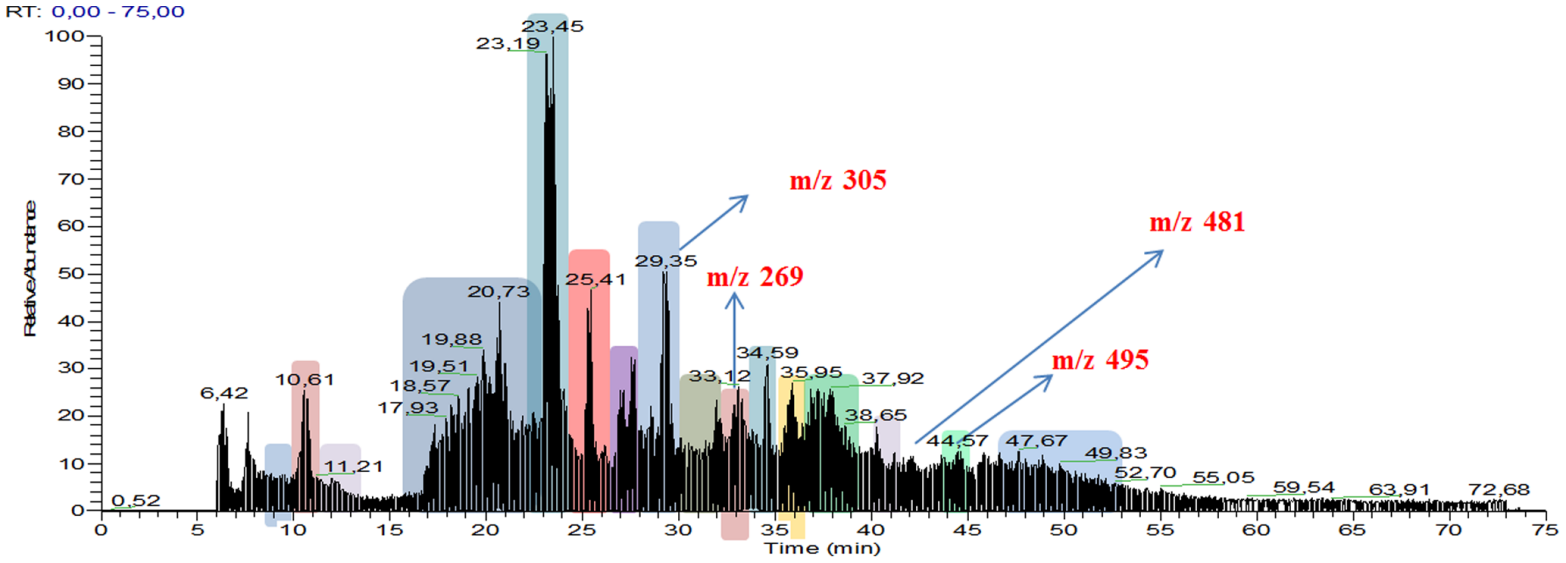
Typical spectra of representative estrogen metabolites, which were obtained in a single injection. The m/z of the molecules and the respective retention time at which they appear are highlighted.

**Figure 4:**
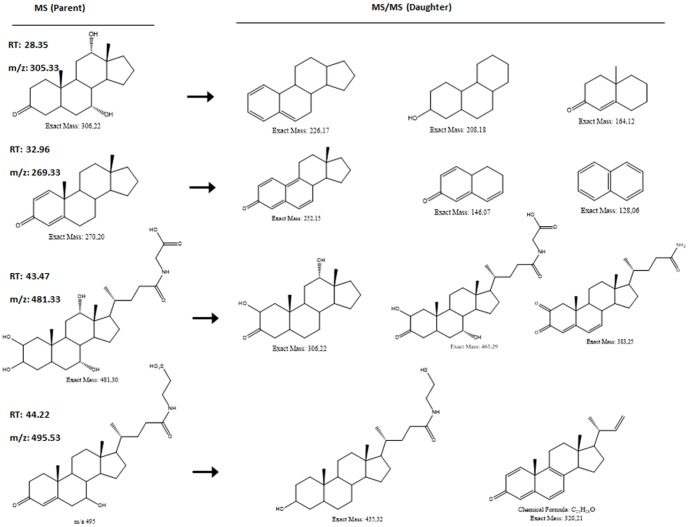
Chemical structures, retention times (RT) and m/z values for the four main components identified by LC–MS analysis of urine of *Schistosoma haematobium*-infected females. Atomic mass (m); atomic charge (z).

Analysis of urine was undertaken using by LC/DAD/ESI-MSn. Inspection of the chromatography revealed several intense peaks, and four of these peaks corresponded to novel metabolites with catechol-estrogen-quinone radicals that, to our knowledge, have not been described previously. We analyzed the estrogen-related compounds using LC/DAD/ESI-MSn to yield the typical intensity spectra of the representative estrogen derivatives obtained by LC (Fig. 3). Each metabolite was detected and identified based on the parameters that were ostensibly unique to it, including the m/z value (m: atomic mass; z: atomic charge) and the retention time (RT). This produced the ‘parent’ mass spectrum by MS (parent m/z). Four new molecules were identified in *S. haematobium*-positive participants, at 28.35, 32.96, 43.47 and 44.22 RT (time at which a peak appeared). The corresponding RT of each peak was submitted to LC/DAD-ESI/MS and revealed a unique high peak in each -- 305.33, 269.33, 481.33 and 495.33 mz, respectively. These spectra were not observed in urine of non-schistosome infected participants (OR 3.35; 95% CI 2.32–4.84; *P*≤0.01). Thereafter, each of these peaks was submitted to ICD/MS/MS, which produced the mass spectra for the ‘daughter’ ions (daughters m/z) (Fig. S1). The collision at high pressure fragments the molecule at its most fragile sites, releasing several smaller molecules. Values of the RT, parent m/z, daughter m/z and the molecular mass of the four new catechols are provided in the Table S1. The chemical structure of each metabolite was reconstructed by overlaying the structures of the m/z's of daughter ions to ascertain whether they corresponded to the structure of each parent m/z (Fig. 4). In each case, full confirmation of the structural assignment for the four novel estrogen-like metabolites was established.

### Estrogen metabolites of schistosomes in urine: an association with self-reported infertility

Comparisons between *S. haematobium* +ve and *S. haematobium* –ve participants revealed a significant association between presence of *S. haematobium* eggs in the urine and hematuria and presence of estrogen metabolites in urine and Group 2 (self-reported primary infertility) (Table 3). A significant association between self-reported infertility (Group 2+3) and *S. haematobium* estrogen metabolites in urine also was observed (Table 4).

**Table 4:** Numbers of positive (E+) and negative (E-) estrogen metabolites in urine samples according to the fertility status of the study participants.

	E + (n = 25)	E - (n = 21)	OR	95% CI	*P*-value
Fertile women (ages)	2 (29, 63)	6 (28–94)			
Group 2+3 (ages)	15 (19–41)	2 (21–34)	4.33	1.13–16.70	0.03
Group 2 (ages)	9 (18–20)	1 (21)	2.67	0.60–11.80	n.a.
Group 3 (ages)	6 (27–41)	1 (34)	4.75	0.51–44.50	n.a.
Total	17	8			
					
≤12 years	8	13			
Total	25	21			

Women unable to become pregnant after one year of trial (Self-reported primary infertility - Group 2) and those who had borne fewer children than desired (Self-reported secondary infertility - Group 3).

OR, odds ratio; CI, confidence interval.

## Discussion

Female genital schistosomiasis (FGS) is common in rural regions of sub-Saharan Africa; of the estimated 70 million children currently infected with *S. haematobium*, approximately 19 million girls and women will develop FGS in the coming decade [24]. Up to 75% of girls and women with chronic *S. haematobium* infection may be affected by deposition of eggs with granulomas and sandy patches on the cervix and, moreover, histopathological interpretation of biopsy specimens indicates that this increases the vascular density of the genital mucosa. [25]. The resulting FGS has been associated with contact bleeding, discharge, pain on intercourse, as well as diminished fertility [26], [27], [28]. FGS can also be a source of shame and stigma [26], [29].

In this cross-sectional study of 93 girls and women, schistosomiasis haematobia was statistically associated with self-reported primary infertility in these participants residing at a rural site, Bengo province, Angola. The outcome corroborated findings from case reports of decreased fertility in women with schistosomiasis [26], [27], [28], [29], [30], [31], [32]. These reports had indicated the need for further investigation given they dealt with only two community-based studies [33], [34]. Travelers who contract schistosomiasis haematobia in endemic regions may be also at risk of infertility [35], [36]. FGS may induce pregnancy-related disorders, but the data available so far are insufficient from which to draw firm conclusions. Functional and anatomical disorders due to schistosome egg granuloma, including fibrosis of the ovaries and tubal obstruction, are established causes of infertility [37]. Hormonal disturbances during FGS may contribute to infertility and sub-optimal fecundity [38]. In this regard, it is relevant to note that *S. haematobium* expresses estrogenic molecules that down regulate the expression of estrogen receptors *in vitro* [11], [13]

Manifestations of FGS in the young are difficult to evaluate because intra-vaginal inspection is only infrequently performed before the onset of puberty and/or sexual activity. Moreover, gynecological examination of people who have lived with these conditions for long periods requires appropriate cultural insight and skill in communication on the part of the investigators [26]. Accordingly, research in rural sites – including this present report -- is challenging, and requires the establishment of a differential diagnosis using non-invasive approaches [26]. Accordingly, development of non-invasive tests for FGS, especially given the potential for FGS- associated infertility, is a worthwhile goal that, if accomplished, can be expected to improve the public health in under-resourced and under-served populations. Based on the present findings, we predict that a diagnostic method based on mass spectrometric analysis of schistosome catechol estrogens in urine of *S. haematobium* infected persons could provide a novel, less invasive tool to complement current approaches.

Here, estrogen-like metabolites similar to those identified previously in adult worm and eggs stages of *S. haematobium* [13], [14] were detected by LC–MS in urine of study participants with FGS, and were statistically associated with self-reported infertility in the study volunteers. These electrophilic compounds can react with DNA to form the depurinating adducts. It is not inconceivable that apurinic sites in chromosomal DNA that result from this type of reaction generate mutations that might underlie infertility. Mutations in genes of the steroid enzyme pathway genes can lead to autosomal recessive infertility [39]. The aromatase enzyme, encoded by the CYP19 gene, converts the androgens testosterone and androstenedione to estradiol and oestrone, respectively. Therefore mutation in CYP19 gene results in estrogen deficiency in both females and males [39]. To date, we have not addressed male infertility caused by *S. haematobium* infection, but we aim to address this aspect in future studies in Bengo [6], [40], [41].

Even though our present report presents a statistical association between the presence of estrogen metabolites in the urine of *S. haematobium*-infected females and sub-fertility, several confounding variables may weaken our hypothesis. First, questions about gynecological complaints and sexual activities in adolescents were asked in the presence of parents; this may have led to a systemic underreporting of complaints and/or sexual activity in some of the participants. Second, primary and secondary infertility due to male-associated factors have not been analysed. Third, we did not have access to information about number of abortions and/or other associated gynecological diseases, or information concerning treatment for schistosomiasis received by the study participants. Given the context of the association between FGS and difficulty in becoming pregnant, the presence of putative mutagenic catechol-estrogens in *S. haematobium* infected persons may have practical implications for development of new approaches to control FGS and its sequelae. We have previously reported novel estrogenic molecules in *S. haematobium* and sera from cases of schistosomiasis haematobia [13], [14]. We now present evidence, also by mass spectrometry, of the detection and characterization of similar molecules in the urine of *S. haematobium* infected people, of which the majority are catechol estrogen quinones.

The genotoxicity of estrogen metabolites might be attributed to oxidation of catechol-estrogens to quinones followed by redox cycling and formation of reactive oxygen species (ROS) that in turn react with DNA [42], [43]. The metabolism of estrogens and the production of depurinating estrogen-DNA adducts as well as the generation of ROS can be implicated in a pathway underlying *S. haematobium*-promoted host cell DNA damage. The mutagenic effect of this estrogen-DNA adduct mediated pathway could explain the statistical association between *S. haematobium* infection and reduced fertility. Given that the genome and transcriptomes of eggs, female and male adult worms of *S. haematobium* is available [44], studies utilizing RNA interference and/or other functional genomic tools to silence components of estrogen catabolism pathways e.g. schistosome estradiol 17beta-dehydrogenase should be informative [45]. To conclude, given that the four novel estrogen-like metabolites identified here in urine were associated with both FGS and with difficulty in becoming pregnant, deeper investigation in larger numbers of infected people and in other geographical regions endemic for *S. haematobium* infection is clearly worthwhile.

## Supporting Information

Figure S1 **Mass spectra of catechol estrogen quinones from** ***Schistosoma haematobium*** **infected individuals by MS (parent m/z) and MS/MS (daughters m/z).** (**A**) m/z 305.33; (**B**) m/z 269.33; (**C**) m/z 481.33 and (**D**) m/z 495.53. The top image corresponds to the m/z of the four estrogen metabolites with catechol-quinone radicals (A–D) found at the retention times (RT) illustrated in Fig. 3 and obtained by LC/DAD-ESI/MS. The maximum peak of each (arrows) was submitted to CID/MS/MS, where the collisions released the mass spectra of the “daughters” (bottom image: encircled). Atomic mass (m); atomic charge (z).
(PDF)

Table S1 **Estrogen metabolites with spectrometric masses of parent and daughter ions and retention times.**
(DOCX)
