# Supplementary material for: Urinary Estrogen Metabolites and Self-Reported Infertility in Women Infected with Schistosoma haematobium
Source: PLoS One. 2014 May 21;9(5):e96774. doi: 10.1371/journal.pone.0096774 (PMC4029575; doi:10.1371/journal.pone.0096774)
Supplement: Figure S1 — Mass spectra of catechol estrogen quinones from Schistosoma haematobium infected individuals by MS (parent m/z) and MS/MS (daughters m/z). (A) m/z 305.33; (B) m/z 269.33; (C) m/z 481.33 and (D) m/z 495.53. The top image corresponds to the m/z of the four estrogen metabolites with catechol-quinone radicals (A–D) found at the retention times (RT) illustrated in Fig. 3 and obtained by LC/DAD-ESI/MS. The maximum peak of each (arrows) was submitted to CID/MS/MS, where the collisions released the mass spectra of the “daughters” (bottom image: encircled). Atomic mass (m); atomic charge (z). (PDF) [file pone.0096774.s001.pdf]

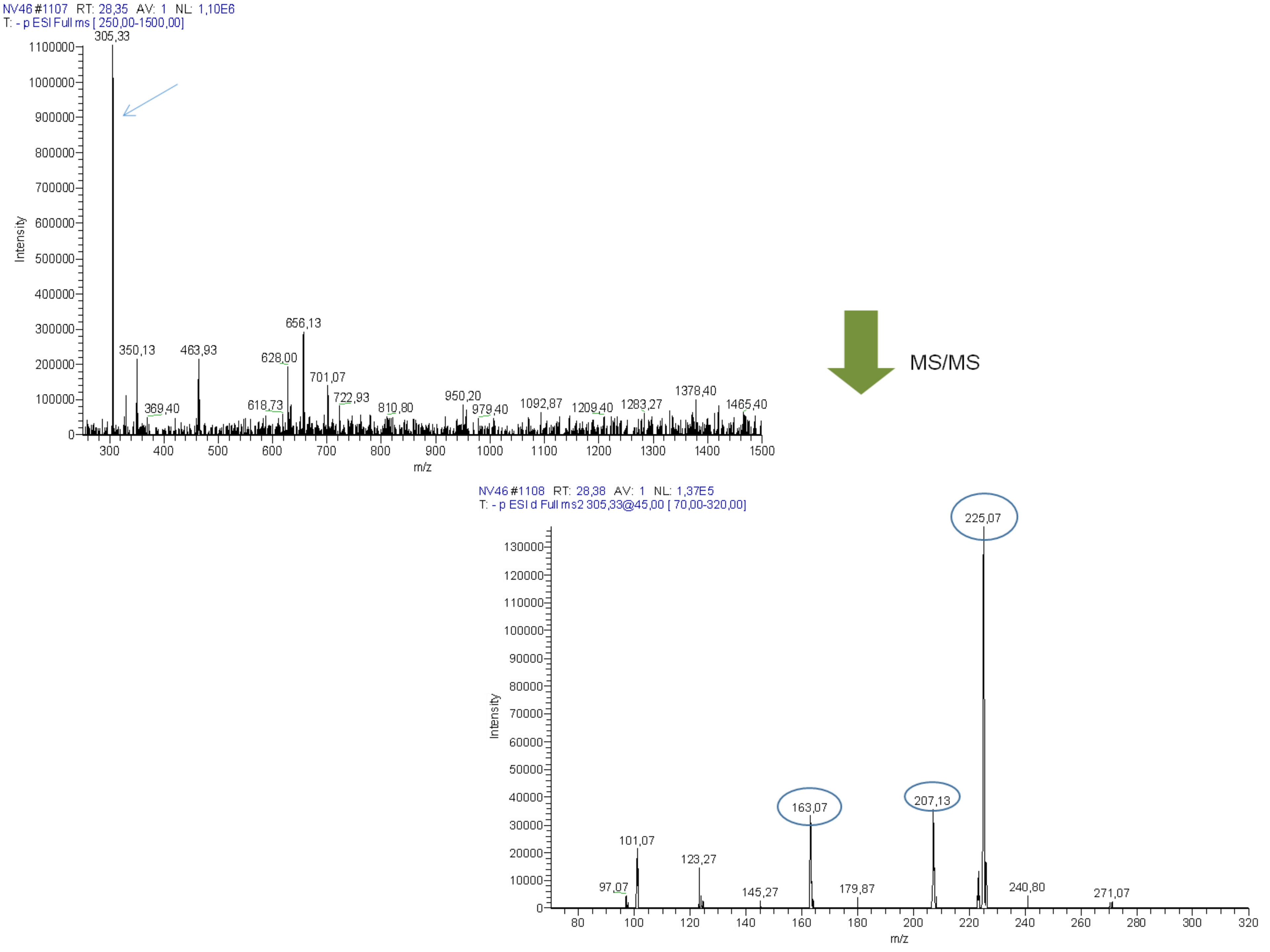
NV46 #1107 RT: 28,35 AV: 1 NL: 1,10E6
T: - p ESI Full ms [250,00-1500,00]
305,33
350,13
463,93
656,13
628,00
701,07
722,93
618,73
369,40
810,80
950,20
979,40
1092,87
1209,40
1283,27
1378,40
1465,40
Intensity
m/z
MS/MS
NV46 #1108 RT: 28,38 AV: 1 NL: 1,37E5
T: - p ESI d Full ms2 305,33@45,00 [70,00-320,00]
225,07
207,13
163,07
101,07
123,27
97,07
145,27
179,87
240,80
271,07
Intensity
m/z

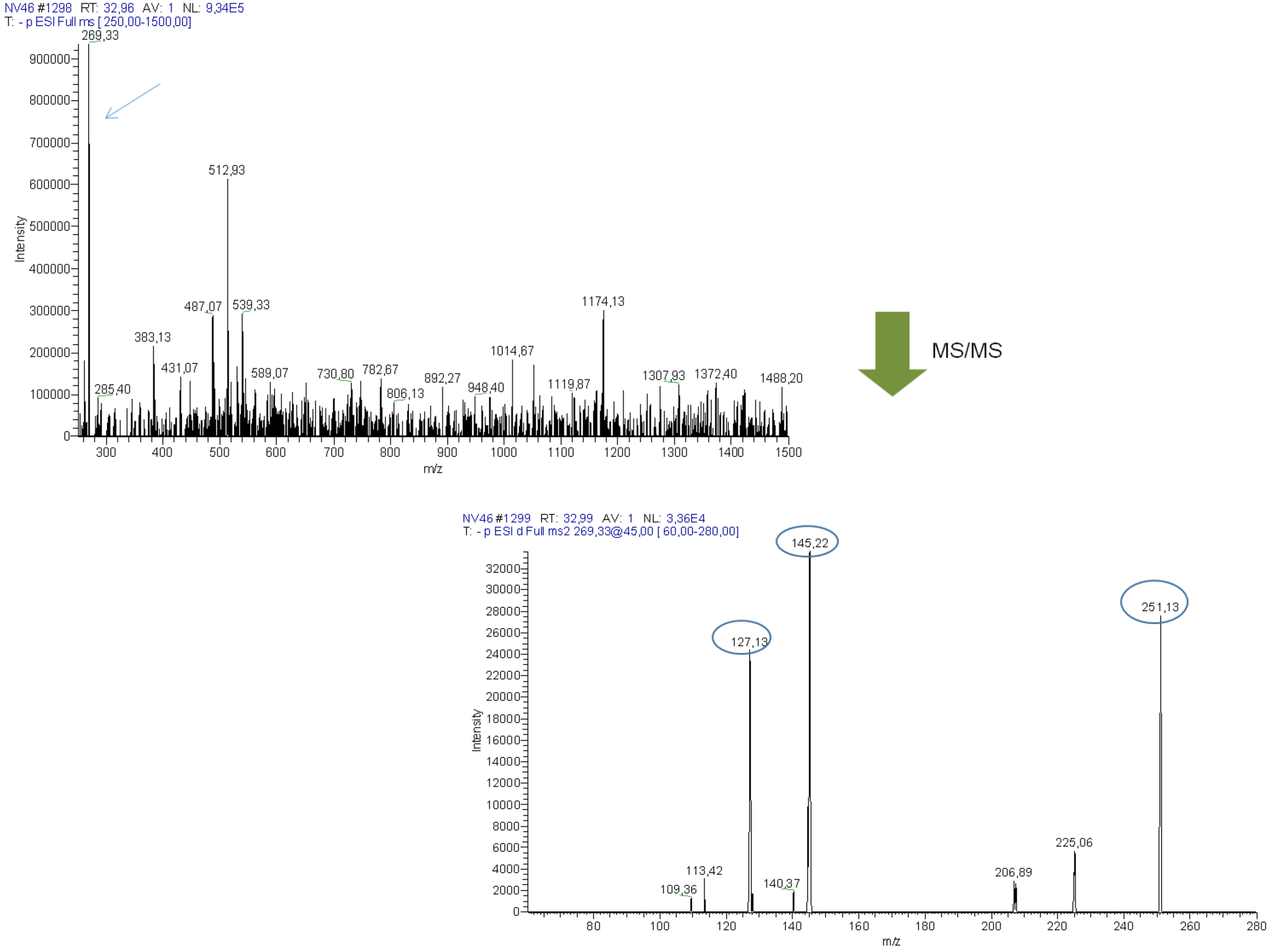

NV46 #1298 RT: 32,96 AV: 1 NL: 9,34E5
T: - p ESI Full ms [250,00-1500,00]
269,33
512,93
487,07
539,33
383,13
431,07
285,40
589,07
730,80
782,67
806,13
892,27
948,40
1014,67
1119,87
1174,13
1307,93
1372,40
1488,20
Intensity
m/z
MS/MS
NV46 #1299 RT: 32,99 AV: 1 NL: 3,36E4
T: - p ESI d Full ms2 269,33@45,00 [60,00-280,00]
145,22
251,13
127,13
113,42
109,36
140,37
206,89
225,06
Intensity
m/z

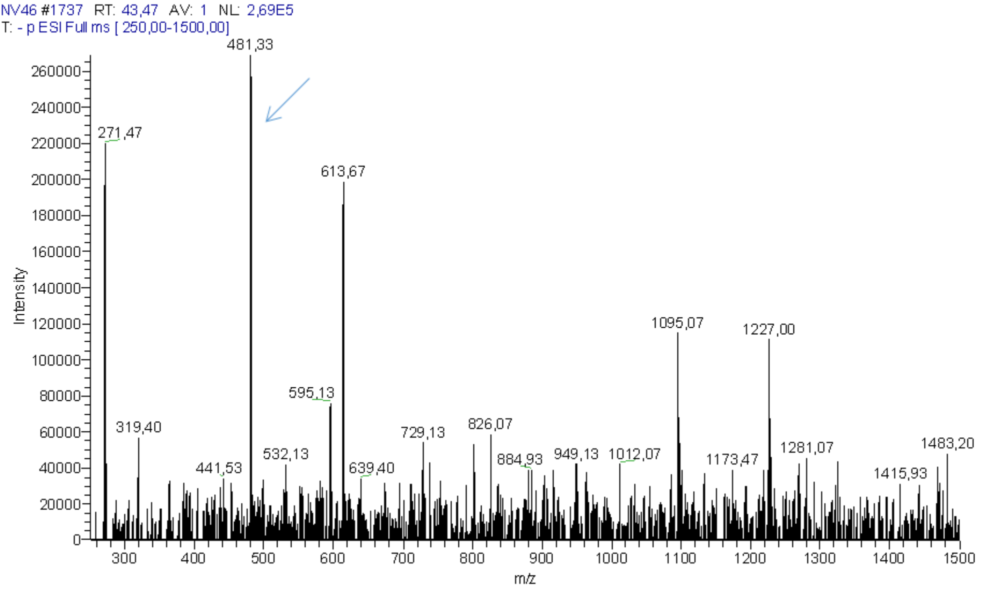
NV46 #1737 RT: 43,47 AV: 1 NL: 2,69E5
T: - p ESI Full ms [250,00-1500,00]
481,33
271,47
613,67
1095,07
1227,00
595,13
319,40
826,07
729,13
532,13
441,53
639,40
884,93
949,13
1012,07
1173,47
1281,07
1415,93
1483,20
Intensity
m/z

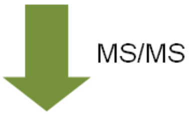
MS/MS

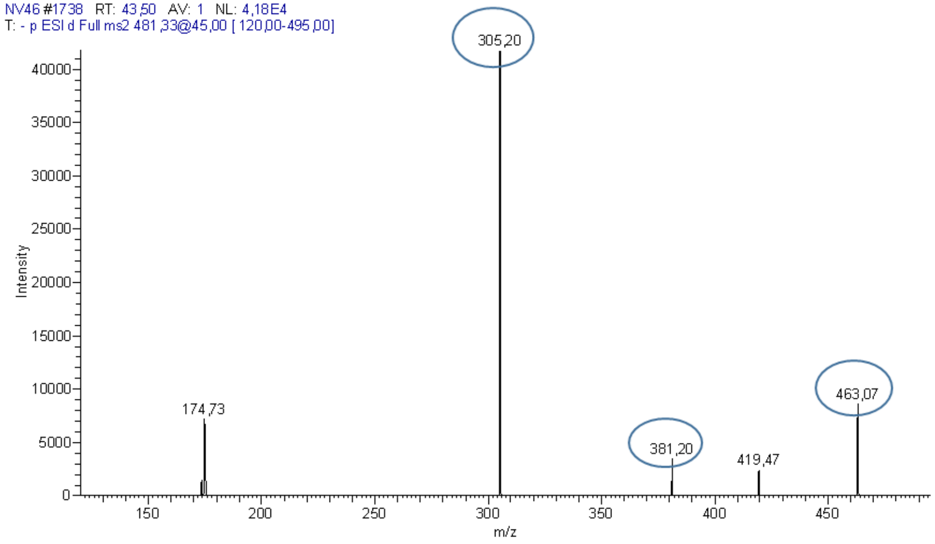
NV46 #1738 RT: 43,50 AV: 1 NL: 4,18E4
T: - p ESI d Full ms2 481,33@45,00 [120,00-495,00]
305,20
174,73
381,20
419,47
463,07
Intensity
m/z

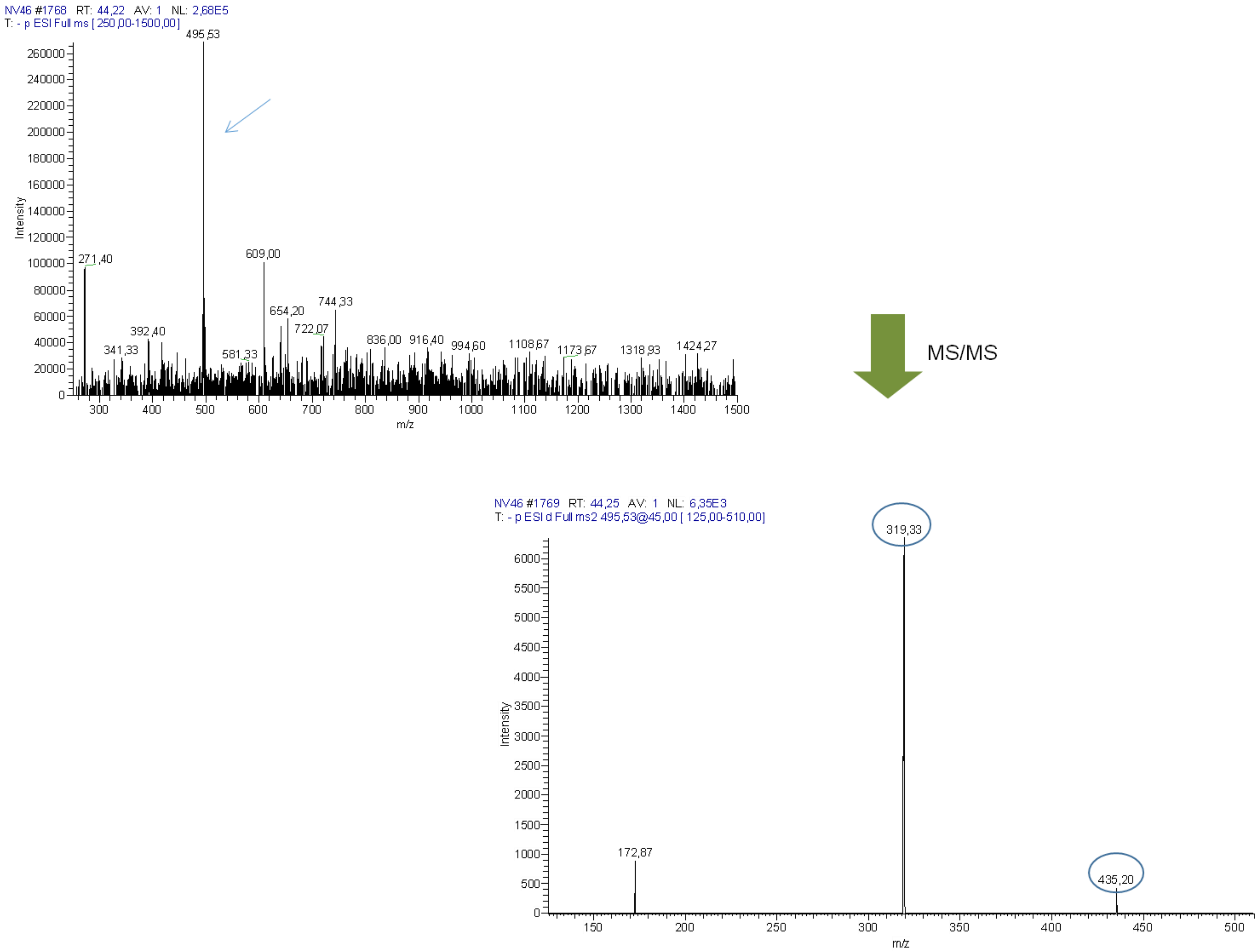
NV46 #1768 RT: 44,22 AV: 1 NL: 2,68E5
T: - p ESI Full ms [250,00-1500,00]
495,53
271,40
609,00
654,20
744,33
722,07
392,40
341,33
581,33
836,00
916,40
994,60
1108,67
1173,67
1318,93
1424,27
Intensity
m/z
MS/MS
NV46 #1769 RT: 44,25 AV: 1 NL: 6,35E3
T: - p ESI d Full ms2 495,53@45,00 [125,00-510,00]
319,33
172,87
435,20
Intensity
m/z
