# Supplementary material for: Urinary Estrogen Metabolites and Self-Reported Infertility in Women Infected with Schistosoma haematobium
Source: PLoS One. 2014 May 21;9(5):e96774. doi: 10.1371/journal.pone.0096774 (PMC4029575; doi:10.1371/journal.pone.0096774)
Supplement: Table S1 — Estrogen metabolites with spectrometric masses of parent and daughter ions and retention times. (DOCX) [file pone.0096774.s002.docx]

**Table S1. Estrogen metabolites with spectrometric masses of parent and daughter ions and retention times.**

| **Compound** | **Mass** | **Parent (m/z)** | **Daughter (m/z)** | **Retention time** | **Classification** |
| --- | --- | --- | --- | --- | --- |
| **1** | 306.22 | 305.33 | 163.07; 207.13; 225.07 | 28.35 | New |
| **2** | 270.20 | 269.33 | 127.13; 145.22; 151.13 | 32.96 | New |
| **3** | 481.30 | 481.33 | 305.20; 463.07; 381.20 | 43.47 | New |
| **4** | 495.00 | 495.53 | 435.20; 319.33 | 44.22 | New |
